# Purinergic Signalling in Physiology and Pathophysiology

**DOI:** 10.3390/ijms24119196

**Published:** 2023-05-24

**Authors:** Ronald Sluyter

**Affiliations:** 1 Molecular Horizons and School of Chemistry and Molecular Bioscience, University of Wollongong, Wollongong, NSW 2522, Australia; rsluyter@uow.edu.au; Tel.: +61-2-4221-5508; 2 Illawarra Health and Medical Research Institute, Wollongong, NSW 2522, Australia

Since its inception by the late Geoffrey Burnstock in the early 1970s [1], the concept of purinergic signalling has grown to encompass a complex network of cell-surface and intracellular organelle P2X, P2Y and P1 (adenosine) receptors, activated by extracellular nucleotides, particularly adenosine 5′-triphosphate (ATP), and adenosine, and various mechanisms of nucleotide and nucleoside metabolism, release, and uptake [2]. Purinergic signalling is now recognized to have important roles in a broad range of physiological and pathophysiological activities, with increasing potential as a therapeutic target in various disorders [3]. To this end, this Special Issue “Purinergic Signalling in Physiology and Pathophysiology” (https://www.mdpi.com/journal/ijms/special_issues/PSPP) (accessed 16 May 2023) in the *International Journal of Molecular Sciences* sought to provide current examples of the various roles of purinergic signalling in a range of physiological and pathophysiological contexts. Along with this editorial, this Special Issue contains five original research articles and nine review articles on this topic.

Seven of the enclosed articles are related to purinergic signalling more broadly. Following an overview of purinergic signalling, Zuccarini et al. [4] provide an extensive review of the roles of purinergic signalling in oral tissues. The distribution of purinergic receptors in the oral cavity and physiological roles of these receptors in the gustatory system, salivary glands, and in teeth and the periodontium are discussed. They also discuss the pathological roles and clinical perspectives of purinergic signalling in relation to oral infections, periodontal disease, Sjogren’s syndrome and radiation damage resulting from the treatment of head and neck cancer.

The review by Vlajkovic and Thorne [5] introduces the cochlea and purinergic signalling. The authors then detail the release of ATP, the distribution of P2X, P2Y and adenosine receptors and ectonucleotidases in the cochlea, and the role of purinergic signalling in cochlea development and noise-, age- and ototoxic-induced hearing loss. Collectively, this article provides a comprehensive overview of purinergic signalling in the cochlea.

Korb et al. [6] provide a systemic review of the role of purinergic signalling in the treatment of COVID-19. This review outlines the findings from the following eight articles: two regarding the P2Y_12_ receptor; two regarding the A_2A_ receptor; one regarding the P2X7, P2Y_14_ and A_3A_ receptors; one regarding the P2X3 receptor and ATP; one regarding CD39 (ecto-nucleoside triphosphate diphosphohydrolase) and CD73; and one regarding ATP. Furthermore, this review outlines the following 13 clinical trials in patients infected with SARS-CoV-2: nine involving P2Y_12_ receptor antagonists, including five regarding clopidogrel; and the remaining four regarding adenosine receptor agonists, antagonists or potentiators. Based on the evidence presented, the authors conclude that drugs targeting the platelet P2Y_12_ receptor remain the most promising medications in those with COVID-19.

The review by Hamoud et al. [7] explores the potential relationship between adenosine, schizophrenia, and cancer. They detail the incidence of cancer in schizophrenia, extracellular adenosine metabolism, adenosine receptor signaling and uric acid metabolism, before outlining the evidence for adenosine system perturbations in schizophrenia, and the role of extracellular adenosine generation in cancer and its potential as a therapeutic target in this disease.

Hu et al. [8] review the roles of CD73 (ecto-5′-nucleotidase) in lung injury. In this review, they present evidence for dual roles for this ectoenzyme in respiratory physiology and pathophysiology. They detail how CD73 can enhance lung injury by promoting cancer and how its blockade may circumvent this disease. They also detail the roles of CD73 in maintaining lung homeostasis and respiratory function, and in protecting the lungs from hypoxic-induced injury. They also discuss the opposing roles of CD73 in lung inflammation.

Volonte et al. [9] provide a highly unique review, in which they introduce *Drosophila* (fruit fly) as an experimental model to study purinergic signalling. This review includes the benefits, barriers and uncertainties using this insect model. This review also details the evidence for the presence of adenosine receptors, and adenosine transporters and metabolic enzymes in *Drosophila*. Notably, this review reinforces the notion that this species appears to lack P2X and P2Y receptors. Collectively, the authors highlight the potential and challenges of *Drosophila* as a model to investigate the physiology and pathophysiology of purinergic signalling.

In the final article related broadly to purinergic signalling, Cicero et al. [10] review dysfunctions of purine metabolism, with a focus on uric acid. Uric acid metabolism is detailed, followed by a review of hyperuricaemia and hypouricaemia, methods of uric acid detection and xanthine oxidoreductase activity, and possible uses of purine metabolites as markers of oxidative stress.

Seven of the enclosed articles focused on a specific member of the purinergic signalling family. The review article by Sophocleous et al. [11] provides an overview of the expression, function, and pharmacology of the P2X4 receptor. This article then details the role of this receptor in neuroinflammatory disorders, such as neuropathic pain, alcohol use disorders, multiple sclerosis and Parkinson’s disease, and its potential as a therapeutic target in these conditions.

De Salis et al. [12] review the gene and protein structures of the human and murine P2X7 receptors, and their isoforms, including an in-depth description of alternative splicing processes and single nucleotide polymorphisms, which give rise to the various P2X7 isoforms. They present a detailed table of 14 human P2X7 isoforms, providing a valuable resource for further studies. They also discuss the pathological implications of P2X7 isoforms in relation to cancer (adenocarcinoma of the lung, neuroblastoma, osteosarcoma, cervical cancer, melanoma, glioblastoma multiforme and leukaemia), Huntington’s disease, and inflammation.

Using the non-selective P2X7 receptor agonist, 2′(3′)-O-(4-benzoylbenzoyl) ATP, and the selective P2X7 antagonists, A-439079 and JNJ-47965567, in mice and rats, as well as *P2rx7* gene knockout mice, this original research article by Zhao et al. [13] confirms a role for P2X7 in the development of depressive-like behaviours in acute and chronic stress. Notably, these pharmacological agents acted in a differential manner in mice and rats, which was attributed to differences in the relative potencies of these compounds between species. Immunohistochemistry revealed that the P2X7 receptor was increased in microglia, but not astrocytes from stressed animals. Based on the electrophysiology recordings of hippocampal astrocytes from these animals, as well as the use of the astrocyte toxin L-α-aminoadipate and the microglial inhibitor minocycline in vivo, it was revealed that microglia induce depressive-like behaviours to relatively mild forms of stress, with astrocytes involved in instances of more pronounced stress.

In their original research article, Hutteau-Hamel et al. [14] investigated the amount of cell-surface P2X7 receptors, as well as plasma membrane GM1 gangliosides (GM1) and cholesterol, in naïve and effector/memory CD4^+^ and CD8^+^ T cells from healthy mice. Whilst the cholesterol amounts were the same between the total CD4^+^ and CD8^+^ T cells, the amounts of P2X7 receptors and GM1 were greater in CD8^+^ T cells compared to CD4^+^ T cells. Furthermore, the P2X7 receptor, GM1 and cholesterol amounts were greater in effector/memory (CD44^high^CD45RB^high^) than naïve (CD44^low^CD45RB^high^) subsets. Cholesterol content correlated with ATP-induced pore formation, phosphatidylserine exposure, and death, but negatively correlated with ATP-induced CD62L shedding in CD4^+^ T cells. In contrast, cholesterol content negatively correlated with each of these ATP-induced responses in CD8^+^ T cells. In general, the enrichment of membrane cholesterol in vitro (using methyl-β-cyclodextrin/cholesterol complexes) or in vivo (through a high-fat diet) lead to a reduction in ATP-induced pore formation, CD62L shedding, and death in CD4^+^ and CD8^+^ T cells, with more complex effects observed at the naïve and effector/memory subset level. Together, these data show that plasma membrane cholesterol content can alter P2X7 receptor-mediated responses in CD4^+^ and CD8^+^ T cells, but that this effect can differ between subsets, highlighting the complexity of P2X7 receptor activation in T cells.

Using blood samples from people with Alzheimer’s disease or mild cognitive impairment and from healthy individuals, Li et al. [15] report in their original research article that amounts of cell-surface P2X7 receptors are decreased on leukocytes from people positive for beta-amyloid plaques. This was observed for classical, non-classical and intermediate monocytes, as well as neutrophils, natural killer cells, and B and T cells combined. This association was also reported for non-classical monocytes and neutrophils from a validation cohort. The reduction in P2X7 receptors corresponded with the reduced numbers of CD11b and CD11c on monocytes and neutrophils. Moreover, reduced P2X7 receptors, CD11b and CD11c were observed at the pre-clinical stage of Alzheimer’s disease and remained reduced throughout disease progression. *P2RX7* and *P2RX4* gene sequencing revealed no association between 12 single nucleotide polymorphism and Alzheimer’s disease in two large cohorts, suggesting that changes in leukocyte P2X7 receptors in this disease are due to environmental not genetic factors.

In their original research article, Nishiyama et al. [16] use an existing database to reveal that *P2RY6* mRNA expression is increased in people with non-alcoholic steatohepatitis (NASH). Moreover, this expression correlated with the expression of *CCL2* and *COL1A1* mRNA, which code for CCL2 and collagen type 1 alpha 1 chain, respectively. The authors further observed that liver *P2ry6* mRNA expression was elevated in a murine model of NASH, which correlated with *Ccl2* mRNA expression. However, NASH development, including liver histology and inflammation, was similar between wildtype and *P2ry6* gene knockout mice. As such, the authors concluded that despite the increased P2Y6 receptors in NASH, this receptor may not contribute to disease.

Finally, in their original research article, Campos-Contreras et al. [17] use an existing database to reveal that the increased expression of *ADORA2B* mRNA, which codes for the A_2B_ receptor, is associated with improved prognosis in ovarian cancer patients at early stages. The authors then demonstrate the presence of *ADORA2B* mRNA and the A_2B_ receptor in the human ovarian adenocarcinoma cell line, SKOV-3. The use of an A_2B_ receptor agonist, BAY-606583, and antagonist, PSB-603, and *ADORA2B* gene knockdown revealed that activation of this receptor impairs SKOV-3 cell migration. This effect of A_2B_ receptor activation was further supported by cDNA microarray analysis, with downregulation and upregulation of pathways such as those associated with cell migration and cell adhesion, respectively.

In conclusion, this Special Issue provides examples of the role of purinergic signalling in various physiological and pathophysiological contexts. Through these examples, the functional breadth of this pathway in health and disease is illustrated in part, continuing to build on the work of Burnstock and many others since the inception of purinergic signalling approximately 50 years ago.
